# Altered Metabolism of the Microbiota–Gut–Brain Axis Is Linked With Comorbid Anxiety in Fecal Recipient Mice of Myasthenia Gravis

**DOI:** 10.3389/fmicb.2022.804537

**Published:** 2022-05-03

**Authors:** Hanping Zhang, Yifan Li, Peng Zheng, Jing Wu, Yu Huang, Xunmin Tan, Xi Hu, Lu Wen, Peijun Xie, Xingyu Zhou, Gang Yu, Libo Zhao, Chanjuan Zhou, Liang Fang, Peng Xie

**Affiliations:** ^1^Department of Neurology, The First Affiliated Hospital of Chongqing Medical University, Chongqing, China; ^2^NHC Key Laboratory of Diagnosis and Treatment on Brain Functional Diseases, The First Affiliated Hospital of Chongqing Medical University, Chongqing, China; ^3^Department of Neurology, Yongchuan Hospital of Chongqing Medical University, Chongqing, China

**Keywords:** myasthenia gravis, anxiety, microbiota, gut-brain axis, metabolism

## Abstract

Myasthenia gravis (MG) comorbid anxiety seriously affects the progress of MG. However, the exact relationship remains poorly understood. Recently, our preliminary study has revealed that intestinal microbe disturbance is closely related to MG. Therefore, further exploration of whether the microbiome is involved in MG comorbid anxiety is warranted. In this study, gas chromatography-mass spectrometry metabolomics analysis was used to characterize the metabotype of feces, serum, and three brain regions involved in emotion (i.e., the prefrontal cortex, hippocampus, and striatum), which were obtained from mice that were colonized with fecal microbiota from patients with MG (MMb), healthy individuals (HMb), or co-colonization of both patients and healthy individuals (CMb). Functional enrichment analysis was used to explore the correlation between the “microbiota–gut–brain” (MGB) axis and anxiety-like behavior. The behavioral test showed that female MMb exhibited anxiety-like behavior, which could be reversed by co-colonization. Moreover, metabolic characterization analysis of the MGB axis showed that the metabotype of gut-brain communication was significantly different between MMb and HMb, and 146 differential metabolites were jointly identified. Among these, 44 metabolites in feces; 12 metabolites in serum; 7 metabolites in hippocampus; 2 metabolites in prefrontal cortex; and 6 metabolites in striatum were reversed by co-colonization. Furthermore, the reversed gut microbiota mainly belonged to bacteroides and firmicutes, which were highly correlated with the reversed metabolites within the MGB axis. Among three emotional brain regions, hippocampus was more affected. Therefore, disturbances in gut microbiota may be involved in the progress of anxiety-like behavior in MG due to the MGB axis.

## Introduction

Myasthenia gravis (MG) is a debilitating autoimmune disease characterized by variable muscle weakness and fatigue; the underlying cause is predominantly thought to be B-cell-mediated antibodies that are directed against acetylcholine receptors at the neuromuscular junction (Gilhus and Verschuuren, 2015; Gilhus, 2016). The fluctuating characteristics of the disease and the potential risk of a myasthenia crisis make patients prone to irritability and anxiety (Paradis et al., 1993; Kulaksizoglu, 2007). Moreover, corticosteroid therapy induces adverse psychiatric effects, such as anxiety (Warrington and Bostwick, 2006). Thus, psychiatric comorbidity is common in patients with MG and usually presents as anxiety and depressive disorders. In a recent study, 26.1% of 69 well-characterized patients with MG have been confirmed to have comorbid anxiety (Alekseeva et al., 2019). The complex interaction between MG and anxiety needs to be highlighted because comorbid anxiety disorders can aggravate the clinical course of MG, raise health care costs, and increase mortality and morbidity. Thus, determining the pathological mechanism underlying MG comorbid anxiety is crucial.

The human gastrointestinal tract harbors a dynamic and complex microbial ecosystem that is composed of bacteria, yeasts, archaea, single-celled eukaryotes, and helminthic parasites, as well as viruses, such as bacteriophage, which live symbiotically with the humans and impact the immune function and metabolism of the host (Nelson et al., 2010; Almeida et al., 2021). Previous evidence has shown that gut microbiome dysfunction is linked with mental disorders, such as stroke, epilepsy, Parkinson’s disease, depression, anxiety, and schizophrenia, as well as various complex diseases, such as obesity, diabetes, and cancer, *via* the “microbiota–gut–brain” (MGB) axis (Wen et al., 2008; Henao-Mejia et al., 2012; Benakis et al., 2016; Sampson et al., 2016; Sharon et al., 2016; Olson et al., 2018; Singh et al., 2018; Zheng et al., 2019b). Recently, it has become increasingly evident that the gut microbiome is a crucial regulator of host immune homeostasis (Levy et al., 2017; Zitvogel et al., 2018), and its dysregulation may mediate the development and diagnosis of both central (e.g., multiple sclerosis) and peripheral (e.g., rheumatoid arthritis) autoimmune diseases (Zhang et al., 2015; Hindson, 2017). In our previous preliminary study, we verified the interaction between perturbed microbial ecology and MG (Zheng et al., 2019a). Notably, germ-free (GF) mice lacking gut microbiota have been shown to exhibit greater anxiety-like behavior than normal specific pathogen-free (SPF) mice, which led to the hypothesis that gut microbiota is a potential cause of MG comorbid anxiety.

To answer the above questions, we sought to explore the metabolic mechanism of the microbiome acting on the MGB axis that leads to comorbid anxiety in MG. Both sexes of GF mice, which underwent fecal microbiota transplantation (FMT) of microbiota from patients with MG, healthy controls, and both patients and controls, were studied to examine whether disturbances in gut microbiota in MG cause comorbid anxiety (Sampson et al., 2016; Sharon et al., 2019). MG is known as neuromuscular junction disease and has no association with emotional management centers of the brain; thus, we analyzed the metabolic alternation of feces, serum, and three emotional management centers of the brain (i.e., the hippocampus, prefrontal cortex, and striatum) (Chiavegatto et al., 2009; Adhikari et al., 2010; Becker et al., 2017; Hare and Duman, 2020; Karayol et al., 2021).

## Materials and Methods

### Animals and Sample Collection

**FIGURE 1:**
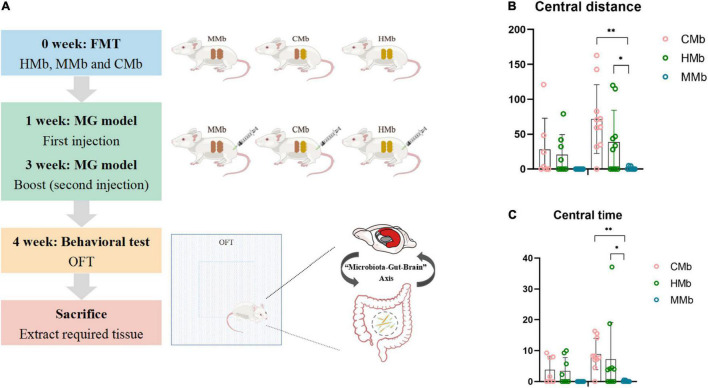
Flow chart and behavioral performance of the experiment. **(A)** Flow chart of the experiment. **(B,C)** Central motion distance and central activity time in both genders among CMb, HMb, and MMb. Notably, compared with female mice in the HMb group, central motion distance (*p* = 0.035) and central activity time (*p* = 0.022) in female MMb was lower, and this difference could be reversed by co-colonization (central motion distance, *p* = 0.000; central activity time, *p* = 0.005). *p < 0.05, ^**^p < 0.01. FMT, fecal microbiota transplantation; MG, myasthenia gravis. MMb, GF mice (*n* = 15) were initially colonized with gut microbiota from MG patients (male/female = 7/8); HMb, GF mice (*n* = 18) were colonized with gut microbiota from healthy individuals (male/female = 8/10); CMb, GF mice (*n* = 17) were simultaneously colonized with gut microbiota from both MG patients and healthy individuals (male/female = 7/10). M, male; F, female.

This research was approved by the Ethics Committee of both Chongqing Medical University and Army Medical University and performed according to the National Institutes of Health Guide for the Care and Use of Laboratory Animals. The entire workflow was that which was used in our previous study and is presented in Figure 1A (Zheng et al., 2019a). Briefly, fecal samples were collected from patients with MG (*n* = 8) and healthy individuals (*n* = 8). Details of donors are provided in Table 1. Each 100 mg fecal sample was diluted in 1.5 ml of reduced sterile phosphate-buffered saline and divided into equal volumes. The GF mice, which were provided by the Department of Laboratory Animal Science of the Third Military Medical University, Chongqing, China, were given the above liquid fecal suspension by gavage.

**TABLE 1:** Detailed demographic information of the donors.

	MG	HC	*P*-value
Sample size	8	8	/
Age (years)^a^	43.0 ± 15.3	43.1 ± 13.9	0.985
Gender (M/F)^b^	2/6	2/5	0.334
Duration (years)	5.0 ± 3.9	/	/
QMGS*^c^*	10.5 ± 8.7	/	/
HAMA (physician—evaluated)	6.13 ± 4.11	/	/
History of respiratory crisis	8	/	/
Immunosuppressive treatment	8	/	/
Clinical phenotypes of MG			
GMG	8	/	/
OMG	/	/	/

*MG, myasthenia gravis; HC, healthy control. OMG, ocular myasthenia gravis; GMG, generalized myasthenia gravis.*

*^a^Two-tailed student test for continuous variables (age).*

*^b^Chi-square analyses for categorical variables (sex).*

*^c^Quantitative Myasthenia Gravis (QMG) test. QMG scores range from 0 to 39. All continuous variables are expressed as the Mean ± SE.*

To assess how disturbances in the microbiome influence the development of anxiety traits in MG, the FMT experiments were performed in both male and female GF mice. One group of GF mice was colonized with gut microbiota from patients with MG (MMb; *n* = 15, male/female = 7/8). As a control, another group of GF mice was colonized with gut microbiota from healthy individuals (HMb; *n* = 18, male/female = 8/10). All MMb and HMb mice were bred in separate autoclaved microisolators with mutually independent sterile air supplies, and all usual supplies were in accord with sterilized standards to prevent internalization of the gut microbiota. In addition, to further verify the effect of gut microbiota, the co-colonization recipient mice were simultaneously colonized with fecal contents from both patients with MG and healthy controls (CMb; *n* = 17, male/female = 7/10).

One week after FMT, all mice were molded with the classic MG model (i.e., the experimental autoimmune MG mouse model) (Li et al., 2016). As described in our previous study, both hind footpads and the backs of all mice were first subcutaneously injected with 200-μl inoculums, containing 50-μg R97-116 peptide [DGDFAIVKFTKVLLDYTGHI, synthesized by Sangon Biotech (Shanghai) Co., Ltd., Shanghai, China] and complete Freund’s adjuvant (Sigma-Aldrich). Two weeks after the first injection, a second booster injection was administered to the same site using a 100-μl mixture. A further week later, all the mice underwent behavioral testing. All the mice were then sacrificed, and the tissues were collected and stored at −80°C for metabolite detection.

### Behavioral Testing

The open-field test (OFT), which consisted of a square base (45 cm^2^ × 45 cm^2^) with black 45-cm high walls, was used to evaluate whether gut microbiota influences the anxious behavior of the mice. All the mice were first transferred to the test room for acclimation for at least 1 h. Each mouse was gently placed in the corner of the equipment to start the behavioral test. After 1 min of adaptation, all spontaneous activity was video recorded for 5 min. The recordings were analyzed using a video-computerized tracking system (SMART, Panlab, Barcelona, Spain). The total motion distance was an indicator of acting ability, and the central motion distance was defined as an index of anxious behavior.

### Gas Chromatography-Mass Spectrometry

Gas chromatography-mass spectrometry metabolomics analysis was used to characterize the metabolic profiles of feces, serum, and three brain regions (i.e., the prefrontal cortex, hippocampus, and striatum), which were obtained from the MMb, HMb, and CMb groups. Orthogonal partial least square-discriminant analysis (OPLS-DA) was performed to visually differentiate the samples between MMb, HMb, and CMb groups using the SIMCA software (version 14.0, Umetrics, Umea, Sweden), and the quality of the model was described by the R2Y and Q2 values. If the difference between R2Y and Q2 is less than 0.3, and Q2 is greater than 0.5, the model predicts well. Metabolites with a variable importance plot (VIP) score of > 1. and a significantly different expression between MMb and HMb groups [false discovery rate (FDR) < 0.05] were considered differential metabolites. Differential metabolites that differed between MMb and HMb groups that were similarly expressed across HMb and CMb groups were considered reversed metabolites. Pathway enrichment analysis was carried out using the MetaboAnalyst^1^ and Kyoto Encyclopedia of Genes and Genomes databases (KEGG).^2^

### Statistical Analysis

Statistical analysis was performed using SPSS 20.0 (Chicago, United States). Continuous variables (e.g., age and behavioral data) are presented as means ± standard errors and were analyzed using Student’s *t*-test or a one-way ANOVA. Categorical data (e.g., sex) were analyzed using a chi-square test. Spearman correlation was used to describe the mutual relationship of a single substance, and the Mantel test was used to test the correlation between two types of variables. A *p* < 0.05 was considered statistically significant. Data were visualized using R (v 4.1.0) and GraphPad Prism 8.0 (San Diego, California, United States).

## Results

### Female Myasthenia Gravis Microbiota Recipient Mice Exhibit Anxiety-Like Behavior

The patients with MG and healthy controls did not differ significantly in age (*p* = 0.985, Student’s *t*-test) or sex proportion (*p* = 0.334, Student’s *t*-test). After FMT and induction of the MG model, the motor capacity in the MMb group showed significantly greater reduction compared with that in the HMb group, irrespective of sex, which is presented in our previous study (Zheng et al., 2019a). Notably, except for the existing difference in MG-like behavior, compared with female mice in the HMb group, central motion distance (*p* = 0.035, one-way ANOVA and *post-hoc* test; Figure 1B) and central activity time (*p* = 0.022, one-way ANOVA and *post-hoc* test; Figure 1C) in female MMb were lower, and this difference could be reversed by co-colonization (central motion distance, *p* = 0.000; central activity time, *p* = 0.005; one-way ANOVA and *post-hoc* test). However, the same result was not observed in male mice in the MMb group. These results suggested that dysfunction of the gut microbiome in MG caused comorbid anxiety, albeit in female mice only.

### Overview of the Reversed Metabolites in the Microbiota–Gut–Brain Axis

**FIGURE 2:**
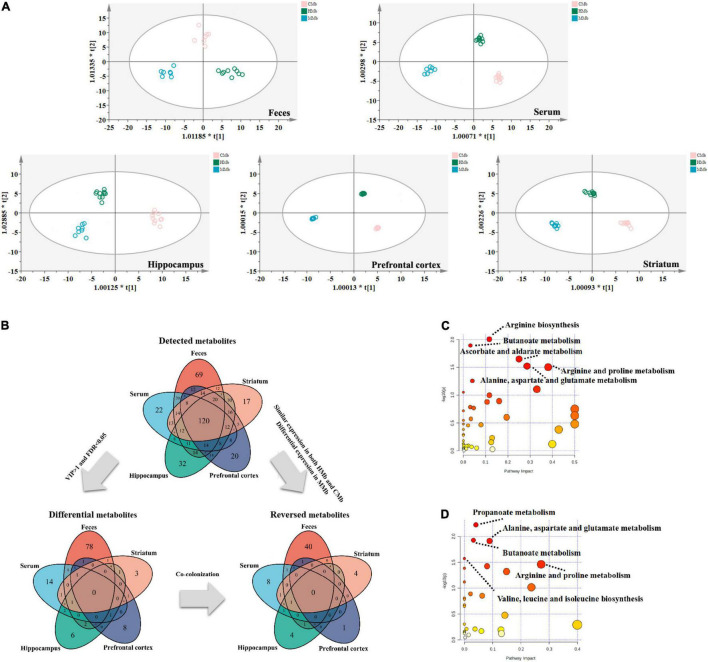
Overview of reversed metabolites in the MGB axis of MG comorbid anxiety. **(A)** Orthogonal partial least square discriminant analysis (OPLS-DA) of metabolic status in feces (R2X[cum] = 0.353, R2Y[cum] = 0.944, Q2[cum] = 0.711), serum (R2X[cum] = 0.5, R2Y[cum] = 0.986, Q2[cum] = 0.842), hippocampus (R2X[cum] = 0.498, R2Y[cum] = 0.954, Q2[cum] = 0.643), prefrontal cortex (R2X[cum] = 0.74, R2Y[cum] = 0.999, Q2[cum] = 0.742) and striatum (R2X[cum] = 0.638, R2Y[cum] = 0.989, Q2[cum] = 0.778) in MGB axis showed clear discrimination among CMb, HMb, and MMb groups. **(B)** Total amount of detected metabolites, differential metabolites and reversed metabolites from feces, serum, hippocampus, prefrontal cortex and striatum found in HMb and MMb groups. **(C)** The metabolic pathways in which all differential metabolites were enriched. **(D)** The metabolic pathways in which all reversed metabolites were enriched. MGB axis, “microbiota-gut-brain” axis.

To examine metabolic dysfunctions within the MGB axis that is involved in MG comorbid anxiety, the reversed metabolites belonging to feces, serum, and three brain tissues (i.e., hippocampus, prefrontal cortex, and striatum) were analyzed. OPLS-DA of the metabolic profiles of these brain tissues in the gut–brain axis showed clear discrimination among CMb, HMb, and MMb groups (Figure 2A). An overview of the reversed metabolites in the MGB axis of MG comorbid anxiety is provided in Figure 2B. We detected 365 metabolites in feces, 290 metabolites in serum, 319 metabolites in the hippocampus, 290 metabolites in the prefrontal cortex, and 298 metabolites in the striatum, and 120 of the above metabolites were expressed in all samples. Using the criterion of FDR < 0.05 and VIP > 1 to discriminate differential metabolites between MMb and HMb, 88 metabolites in feces, 24 metabolites in serum, 13 metabolites in the hippocampus, 12 in the prefrontal cortex, and 9 metabolites in the striatum were selected as differential metabolites; however, there are no common differential metabolites in feces, serum, and three brain regions involved in emotion (i.e., the prefrontal cortex, hippocampus, and striatum). The top three metabolic enrichment pathways of all the differential metabolites were arginine biosynthesis, butanoate metabolism, and ascorbate and aldarate metabolism (Figure 2C).

Of all the above differential metabolites that discriminated between MMb and HMb, those that were similarly expressed in both HMb and CMb were defined as reversed metabolites. In MMb, there were 44 reversed metabolites (44/88, 50.0%) in feces, of which 26 were upregulated and 18 were downregulated; 12 reversed metabolites (12/24, 50.0%) in serum, of which six were upregulated and six were downregulated; 7 reversed metabolites (7/13, 53.8%) in the hippocampus, of which one was upregulated and six were downregulated; 2 reversed metabolites (2/12, 16.7%) in the prefrontal cortex, of which two were upregulated; and 6 reversed metabolites (6/9, 66.7%) in the striatum, of which one was upregulated and five were downregulated. The top three metabolic pathways in which the whole reversed metabolites were involved were propanoate metabolism, butanoate metabolism, and alanine, aspartate, and glutamate metabolism (Figure 2D). Further details are presented in Supplementary Tables 1, 2.

These results indicated that the metabolic effects of gut microbes along the gut–brain axis may be tissue specific. Furthermore, in contrast to the metabolic characteristics of the MMb group, the metabotype of the CMb group, which was induced by the mutual communication of the microbiome between the patients with MG and healthy controls, was partly reversed.

### Metabolic Pathways of the Reversed Metabolites in the Feces and Serum With the Microbiota–Gut–Brain Axis

**FIGURE 3:**
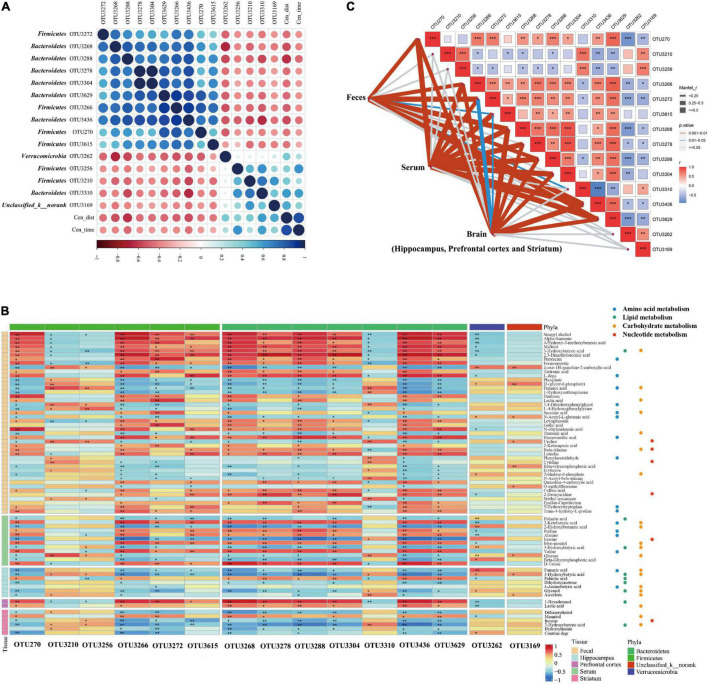
Correlation between the abundance of reversed operational taxonomic units (OTUs) closely associated with anxiety-like behavior and reversed metabolites in the MGB axis. **(A)** Heatmap of correlation between the abundance of reversed OTUs and anxiety-like behavior. Fifteen OTUs were found closely related to anxious behavior, most of which were concentrated in Firmicutes (OTU270, OTU3210, OTU3256, OTU3266, OTU3615, and OTU3272) and Bacteroidetes (OTU3268, OTU3288, OTU3436, OTU3278, OTU3304, OTU3629, and OTU3310). Verrucomicrobia (OTU3262) and Unclassified_k_norank (OTU3169) were also involved. **(B)** Heatmap of correlation between the abundance of reversed OTUs closely associated with anxiety-like behavior and reversed metabolites in MGB axis. Reversed metabolic substances had a strong or weak association with reversed gut microbes, although significance was not reached for all. *P < 0.05, **P < 0.01. **(C)** Mantel test of the correlation between the abundance of reversed OTUs and the entire reversed metabolic status of the various components of the gut-brain communication. *P < 0.05, **P < 0.01, ***P < 0.001.

**FIGURE 4:**
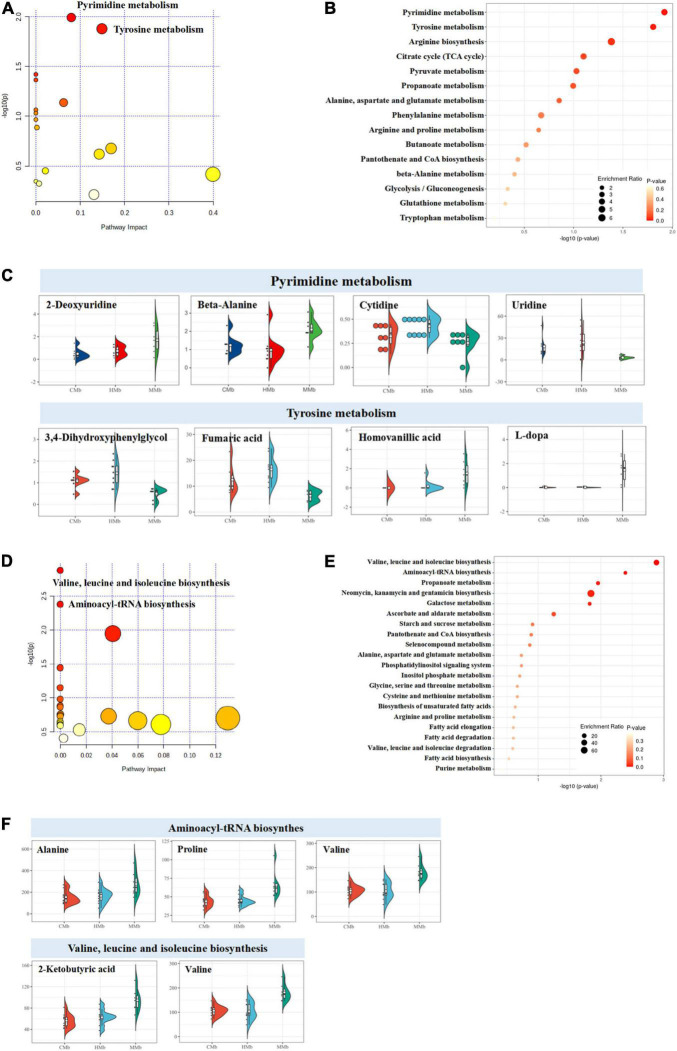
Enriched metabolic pathway analysis of reversed metabolites in feces and serum. **(A–C)** Both the enriched metabolic pathway analysis and the enrichment metabolite set of reversed metabolic profiles in feces. The half violin plot was used to represent the expression of eight reversed substances, which were involved in the top two metabolic pathways: pyrimidine metabolism and tyrosine metabolism. The dots on the left represent the expression of specific metabolites in samples. The size of the dots is related to the degree of dispersion of the overall data. The more discrete the data, the smaller the dots. **(D–F)** The enriched metabolic pathway analysis and enrichment metabolite set of reversed metabolic profiles in serum. Five reversed metabolites were involved in the top two metabolic pathways (valine, leucine, and isoleucine biosynthesis and aminoacyl-tRNA biosynthesis).

Using the KEGG database, in feces, reversed metabolites were largely focused on amino acid metabolism (10 metabolites) and carbohydrate metabolism (seven metabolites) (Figure 3B). Both the enriched metabolic pathway analysis and the enrichment metabolite set of reversed metabolic profiles are shown in Figures 4A,B. The half violin plot was used to represent the expression of eight reversed substances, which were involved in the top two metabolic pathways: Pyrimidine metabolism and tyrosine metabolism (Figure 4C). Cytidine, uridine, 2-deoxyuridine, and beta-alanine were involved in pyrimidine metabolism. L-dopa, 3,4-dihydroxyphenylglycol, homovanillic acid, and fumaric acid were involved in tyrosine metabolism.

In serum, results were the same as those in feces; the reversed metabolic substances were focused on amino acid metabolism (four metabolites) and carbohydrate metabolism (five metabolites) (Figure 3B). The enriched metabolic pathway analysis and the enrichment metabolite set were carried out using the KEGG platform and are illustrated in Figures 4D,E. Five reversed metabolites were involved in the top two metabolic pathways (valine, leucine, and isoleucine biosynthesis and aminoacyl-tRNA biosynthesis) (Figure 4F). Valine and 2-ketobutyric acid were involved in valine, leucine, and isoleucine biosynthesis, and valine, alanine, and proline were involved in aminoacyl-tRNA biosynthesis.

### Reversed Metabolic Profiles Among the Three Emotional Brain Regions Within the Microbiota–Gut–Brain Axis

**FIGURE 5:**
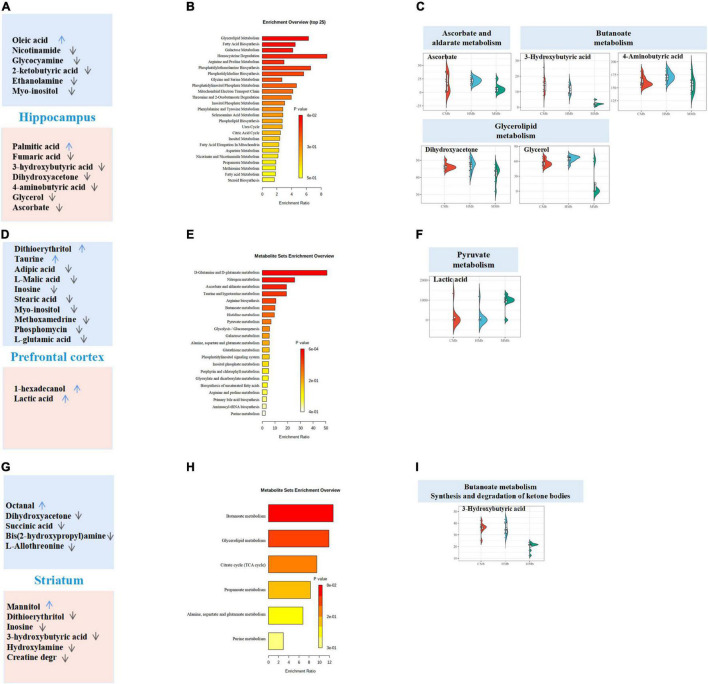
Reversed metabolites enriched in differential metabolic pathways in the three emotional brain. **(A,D,G)** Total differential metabolites between HMb and MMb group in hippocampus, prefrontal cortex, and striatum were, respectively, presented in the boxes. Reversed metabolites among differential metabolites were in the red box, and the remaining differential metabolic substances were in the blue box. The arrow direction indicated more up-regulation or down-regulation of metabolite expression in MMb group compared with HMb group. **(B,E,H)** Enrichment overview of differential metabolites in hippocampus, prefrontal cortex, and striatum. **(C)** Five (71.4%) reversed metabolites were involved in the top three metabolic pathways of the differential metabolic substances in hippocampus. Ascorbate was involved in ascorbate and aldarate metabolism, 4-aminobutyric acid and 3-hydroxybutyric acid were involved in butanoate metabolism, and dihydroxy acetone and glycerol were involved in glycerolipid metabolism. **(F)** Of the top three metabolic pathways of differential metabolic substances in prefrontal cortex, only lactic acid (50.0%) was involved in pyruvate metabolism. None of the reversed metabolites were included in glyoxylate and dicarboxylate metabolism or nitrogen metabolism. **(I)** In striatum, three-hydroxybutyric (16.7%) acid was involved in both butanoate metabolism and the synthesis and degradation of ketone bodies, which were in the top three metabolic pathways of differential metabolic substances. None of the reversed metabolites were involved in glycerolipid metabolism.

The enrichment overview of the differential metabolites between the MMb and HMb in the hippocampus is shown in Figure 5A, where detailed differential and reversed metabolites are presented simultaneously. Apart from the expression of palmitic acid, which was more upregulated, the expression of all other reversed metabolic substances was more downregulated in the MMb group than in the HMb group. Results of the hippocampus contrasted from those of the feces and serum, whereby the reversed metabolites were mostly involved in lipid metabolism (four metabolites) and carbohydrate metabolism (five metabolites) (Figure 3B). Five (5/7, 71.4%) reversed metabolites were involved in the top three metabolic pathways of the differential metabolic substances. Ascorbate was involved in ascorbate and aldarate metabolism (Figures 5B,C), and 4-aminobutyric acid and 3-hydroxybutyric acid were involved in butanoate metabolism, and dihydroxyacetone and glycerol were involved in glycerolipid metabolism.

For the prefrontal cortex, the enrichment overview of the differential metabolites between two groups is presented in Figure 5D. Compared with the HMb group, the expression of both reversed metabolites was more upregulated in the MMb group; 1-hexadecanol was involved in lipid metabolism, and lactic acid was involved in carbohydrate metabolism (Figure 3B). Moreover, of the top three metabolic pathways of differential metabolic substances, only lactic acid (1/2, 50.0%) was involved in pyruvate metabolism (Figures 5E,F). None of the reversed metabolites were included in glyoxylate and dicarboxylate metabolism or nitrogen metabolism.

In regard to the striatum, the enrichment overview of the differential metabolites between the two groups is shown in Figure 5G. Apart from mannitol, the expression of all other reversed metabolic substances was downregulated in the MMb group compared with the HMb group. Two metabolites were involved in carbohydrate metabolism, and all remaining metabolites were involved in lipid metabolism and nucleotide metabolism (Figure 3B). Three-hydroxybutyric acid (1/6, 16.7%) was involved in both butanoate metabolism and the synthesis and degradation of ketone bodies, which were in the top three metabolic pathways of differential metabolic substances (Figures 5H,I). None of the reversed metabolites were involved in glycerolipid metabolism.

In summary, of the three emotional brain regions, the reversed metabolites of the hippocampus contrasted from those of prefrontal cortex and striatum participated to a greater extent in the top three metabolic pathways of differential metabolites.

### Fifteen Reversed Operational Taxonomic Units Were Closely Associated With the Anxiety-Like Behavior of Host Mice

As found previously, 54 reversed OTUs were detected in “MG microbiota” and “healthy controls microbiota” host mice (Zheng et al., 2019a). To clarify the potential relationship between the gut microbiome and MG comorbid anxiety, the correlation between reversed Operational Taxonomic Units (OTUs) and behavioral performance was carried out using Spearman analysis (Figure 3A). We found that 15 OTUs were closely related to anxious behavior, most of which were concentrated in firmicutes (OTU270, OTU3210, OTU3256, OTU3266, OTU3615, and OTU3272) and bacteroidetes (OTU3268, OTU3288, OTU3436, OTU3278, OTU3304, OTU3629, and OTU3310). Verrucomicrobia (OTU3262) and Unclassified_k__norank (OTU3169) were also involved. Further details are provided in Supplementary Table 3.

### Reversed Gut Microbiota Was Closely Related to the Reversed Metabotype During Gut–Brain Communication

All reversed metabolites of different tissues and reversed OTUs that were highly associated with OFT were included to determine the strength of the mutual association (Figure 3B). Reversed metabolic substances had a strong or weak association with reversed gut microbes, although significance was not reached for all. To further explore these associations, we used the Mantel test to evaluate the correlation between the abundance of reversed OTUs and the entire reversed metabolic status of the various components of the gut–brain communication (Figure 3C). All reversed metabolites belonging to the hippocampus, prefrontal cortex, and striatum were integrated into the metabolic changes of the brain. Notably, firmicutes (OTU270, OTU3266, OTU3615, and OTU3272) and bacteroidetes (OTU3268, OTU3288, OTU3436, and OTU3629) were closely related to the entire reversed metabolic status of feces, serum, and the brain. Bacteroidetes (OTU3278 and OTU3304) were only associated with the reversed metabotype of both serum and the brain. Bacteroidetes (OTU3310) were only related to reversed fecal metabolic profiles. However, firmicutes (OTU3210 and OTU3256), verrucomicrobia (OTU3262), and Unclassified_k__norank (OTU3169) had no significant association with any of the reversed metabotypes of feces, serum, or brain. These results suggest that the reversed gut microbiota is closely related to the reversed metabolites during gut–brain communication and is fully or partially involved in the metabolic changes of different components along the MGB axis.

## Discussion

**FIGURE 6:**
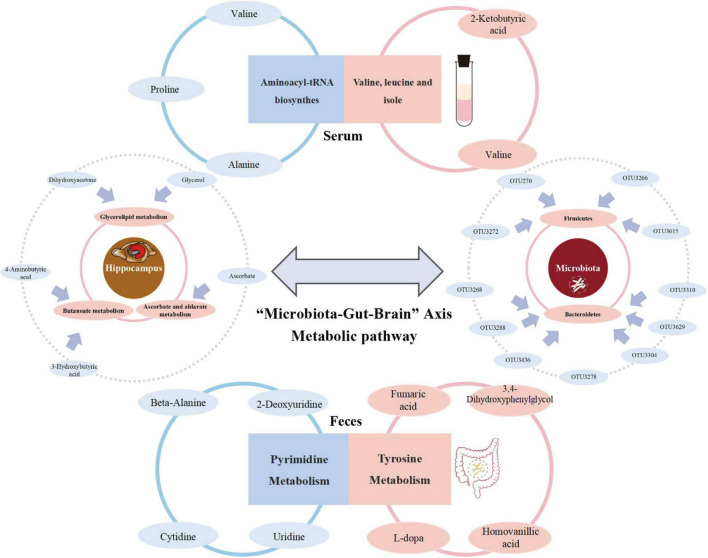
Summary of the metabolism in which reversed metabolites are mainly enriched among “Microbiota-Gut-Brain” Axis during the development of MG comorbid anxiety.

To the best of our knowledge, this is the first research to generate a comprehensive overview of metabolism along the MGB axis of MG comorbid anxiety using GF mice. We not only considered “MG microbiome” and “HC microbiome” recipients but also, importantly, the co-colonization of both types of microbiomes to help identify potential target gut flora and metabolites that could be reversed (Figure 6).

There is preliminary evidence, albeit mostly from animal studies, that suggests potential regulatory effects of microbiota in neuropsychiatric conditions, such as depression and anxiety (Sharon et al., 2016). In this study, we found that disturbances in the intestinal microenvironment in MG may involve the co-existence of specific gut microbiota, and most of them belonged to firmicutes (6/15) and bacteroidetes (7/15), which has the potential to cause an anxiety phenotype. Recent studies have reported that higher bacteroidaceae, bacteroides, lower firmicutes, and ruminococcaceae are detected in participants with generalized anxiety disorder relative to controls (Jiang et al., 2018; Chen et al., 2019; Simpson et al., 2021). Moreover, the reversed OTUs fully or partially interacted with the metabolism of each component along the MGB axis, which suggested that the gut microbiota or its metabolic substances play a role in varying degrees in the process of gut–brain communication. Furthermore, the metabolic response of different tissues along the gut–brain axis, including the feces, serum, and the three emotional brain regions, is specific. Studies have shown that the hippocampus, prefrontal cortex, and striatum are closely related to anxiety (Park and Moghaddam, 2017; Jimenez et al., 2018; Sherwin et al., 2019), which is in line with the results of our study. Differential metabolites identified between the HMb and MMb groups in the three emotional centers were all partially reversed. However, compared with the prefrontal cortex and striatum, the hippocampus had the highest proportion of differential metabolites that could be reversed, of which the reversed metabolites were mostly involved in the top three pathways of differential metabolic substances. It is possible that other significant brain regions also respond to microbiological therapy, which may be an encouraging avenue of future research.

In regard to the mutual interaction of the “MG microbiome” and “HC microbiome,” the metabotype of the CMb group differed from both the MMb and HMb groups to some extent; moreover, its behavioral phenotype was reversed simultaneously, which suggested that gut microbes can effectively influence the brain and behavioral performance *via* the MGB axis. Implementing the CMb group helped identify differential metabolites between the MMb and HMb groups, which could be relatively easily modulated by changes in intestinal microecology. Previous studies have confirmed that the metabolism in which reversed metabolites are mainly enriched is involved in the development of anxiety. Pyrimidine metabolism was shown to be similarly disturbed in the acute coronary syndrome (ACS) with comorbid anxiety compared with the ACS without anxiety (Wei et al., 2021). Tyrosine is closely related to serotonergic activity, which has a close relationship with anxiety (Zhang et al., 2020). Moreover, the reversed metabolic substances of myo-inositol and palmitic acid in the serum of the model mouse were shown to be strongly associated with anxiety-related behaviors (Zhang et al., 2011; Moon et al., 2014; Humer et al., 2020). Preclinical findings have suggested that glycerolipids in the brain play a crucial role in the induction of anxiety and hyperactivity (Müller et al., 2015). However, understanding the specific mechanism requires further investigation.

The incidence of MG comorbid anxiety has a strong female bias (Whitacre, 2001); such a sex bias in anxious behavioral outcomes was observed in the current study, in which female hosts demonstrated evident behavioral disparity. We found that sex influences the diversity and complexity of microbes harboring in the gut. The gut microbiome can directly, indirectly, and reciprocally influence sex steroid hormones and central gene activation, which are critical factors in the process of various mental health disorders, such as depression, anxiety, autism, schizophrenia, anorexia nervosa, Parkinson’s disease, and multiple sclerosis (Jaggar et al., 2020). Animal studies have demonstrated that the female-biased risk of autoimmune disorders is mediated by sex differences in the gut microbiota (Markle et al., 2013). Compared with females to females, the adoptive transfer of gut microbiota from males to females delays the onset and reduces the severity of disease, thus revealing a role for sex-specific microbiome exposure in regulating the features of host behavioral performance. The sex-specific microbiota and the associated mechanism underlying sex–gut–brain differences need further investigation.

The study has several limitations. First, our study included a small number of samples. However, GF mice are relatively rare and costly, and we also studied both sexes; thus, our conclusions are relatively representative and reliable. Second, we only assessed sex differences in anxious behavioral outcomes only. Key sex-specific strains of anxiety traits in MG need to be further identified. Third, detailed metabolic mechanisms and pathways of reversed substances and their specific interactions with brain regions require further clarification in future research.

## Conclusion

Reversed metabolites along the MGB axis found in the HMb, MMb, and HMb groups may help identify specific target microbiota and understand the metabolic mechanism. This will open further avenues for future studies to better understand, diagnose, and treat MG comorbid anxiety.

## Data Availability Statement

The datasets presented in this study can be found in online repositories. The names of the repository/repositories and accession number(s) can be found below: NCBI repository, accession number SUB10579298.

## Ethics Statement

The animal study was reviewed and approved by the Ethics Committee of both Chongqing Medical University and Army Medical University. Written informed consent was obtained from the owners for the participation of their animals in this study.

## Author Contributions

HZ and YL contributed to the investigation, formal analysis, and result visualization. PZ, JW, YH, XT, and XH performed the methodology. LW, PJX, and XZ conducted data curation. GY, LZ, and CZ validated the research details. PX and LF contributed to the conceptualization and supervision of the study. All authors contributed to the article and approved the submitted version.

## Conflict of Interest

The authors declare that the research was conducted in the absence of any commercial or financial relationships that could be construed as a potential conflict of interest.

## Publisher’s Note

All claims expressed in this article are solely those of the authors and do not necessarily represent those of their affiliated organizations, or those of the publisher, the editors and the reviewers. Any product that may be evaluated in this article, or claim that may be made by its manufacturer, is not guaranteed or endorsed by the publisher.

## Footnotes

1
https://www.metaboanalyst.ca/

2
https://www.kegg.jp/
