# Supplementary material for: Altered Metabolism of the Microbiota–Gut–Brain Axis Is Linked With Comorbid Anxiety in Fecal Recipient Mice of Myasthenia Gravis
Source: Front Microbiol. 2022 May 3;13:804537. doi: 10.3389/fmicb.2022.804537 (PMC9111518; doi:10.3389/fmicb.2022.804537)
Supplement: Supplementary file 1 [file Table_1.docx]

**Supplementary table 1. All reversed metabolites in feces, serum, prefrontal cortex, hippocampus and striatum.**

| **Sample** | **Reversed Metabolites** | **HMDB** | **PubChem** | **KEGG** | **Expresion**  **in MMb** | **MMb vs HMb**  **(FDR)** | **CMb vs HMb**  **(FDR)** |
| --- | --- | --- | --- | --- | --- | --- | --- |
| Feces | Sinapyl Alcohol | HMDB0013070 | 5280507 | C02325 | Up | 0.00 | 0.97 |
| Feces | Alpha-Santonin | NA | 221071 | C02206 | Up | 0.00 | 1.00 |
| Feces | 4-Hydroxy-3-Methoxybenzoic Acid | HMDB0000484 | 8468 | C06672 | Up | 0.00 | 0.52 |
| Feces | Maltitol | HMDB0002928 | 493591 | NA | Up | 0.00 | 1.00 |
| Feces | 3-Hydroxybutyric Acid | HMDB0000357 | 441 | C01089 | Up | 0.00 | 0.88 |
| Feces | 2,3-Dimethylsuccinic Acid | HMDB0245405 | 11848 | NA | Up | 0.00 | 1.00 |
| Feces | Putrescine | HMDB0001414 | 1045 | C00134 | Up | 0.00 | 0.60 |
| Feces | Formononetin | HMDB0005808 | 5280378 | C00858 | Up | 0.00 | 0.22 |
| Feces | 4-Oxo-1H-Quinoline-2-Carboxylic Acid | NA | 3845 | NA | Down | 0.00 | 1.00 |
| Feces | Tartronic Acid | HMDB0035227 | 45 | C02287 | Up | 0.00 | 0.39 |
| Feces | L-Dopa | HMDB0000181 | 6047 | C00355 | Up | 0.00 | 1.00 |
| Feces | Phosphate | HMDB0001429 | 57424078 | C00009 | Down | 0.01 | 0.69 |
| Feces | D-(Glycerol-Phosphate) | NA | NA | NA | Down | 0.00 | 0.91 |
| Feces | Fumaric Acid | HMDB0000134 | 444972 | C00122 | Down | 0.00 | 0.27 |
| Feces | 1-Hydroxyanthraquinone | HMDB0243898 | 8512 | C02980 | Down | 0.00 | 0.16 |
| Feces | Daidzein | HMDB0003312 | 5281708 | C10208 | Up | 0.00 | 0.97 |
| Feces | Lactic Acid | HMDB0000190 | 61503 | C00186 | Up | 0.01 | 0.91 |
| Feces | 3,4-Dihydroxyphenylglycol | HMDB0000318 | 91528 | C05576 | Down | 0.00 | 0.18 |
| Feces | L-4-Hydroxyphenylglycine | HMDB0244973 | 582713 | C12323 | Down | 0.03 | 1.00 |
| Feces | Succinic Acid | HMDB0000254 | 1110 | C00042 | Up | 0.01 | 0.11 |
| Feces | N-Acetyl-L-Glutamic Acid | HMDB0001138 | 185 | C00624 | Down | 0.03 | 0.61 |
| Feces | Levoglucosan | HMDB0000640 | 2724705 | C22350 | Up | 0.03 | 0.97 |
| Feces | Gallic Acid | HMDB0005807 | 370 | C01424 | Up | 0.02 | 1.00 |
| Feces | N-Ethylmaleamic Acid | HMDB0255136 | 5380356 | NA | Up | 0.01 | 0.80 |
| Feces | Threonic Acid | HMDB0000943 | 151152 | C01620 | Down | 0.01 | 0.69 |
| Feces | Homovanillic Acid | HMDB0000118 | 1738 | C05582 | Up | 0.01 | 0.80 |
| Feces | Uridine | HMDB0000296 | 6029 | C00299 | Down | 0.03 | 0.61 |
| Feces | 2-Ketocaproic Acid | HMDB0001864 | 159664 | C00902 | Up | 0.02 | 0.77 |
| Feces | Beta-Alanine | HMDB0000056 | 239 | C00099 | Up | 0.02 | 0.61 |
| Feces | Luteolin | HMDB0005800 | 5280445 | C01514 | Up | 0.03 | 0.43 |
| Feces | Phenylacetaldehyde | HMDB0006236 | 998 | C00601 | Down | 0.02 | 0.14 |
| Feces | Cytidine | HMDB0000089 | 6253 | C00475 | Down | 0.02 | 0.14 |
| Feces | Beta-Glycerophosphoric Acid | HMDB0002520 | 2526 | C02979 | Down | 0.03 | 0.95 |
| Feces | Erythrose | HMDB0002649 | 5460672 | C01796 | Down | 0.01 | 0.12 |
| Feces | Trehalose-6-Phosphate | HMDB0001124 | 122336 | C00689 | Down | 0.04 | 0.78 |
| Feces | N-Acetyl-Beta-Alanine | HMDB0061880 | 76406 | C01073 | Down | 0.01 | 0.14 |
| Feces | Quinoline-4-Carboxylic Acid | HMDB0257047 | 10243 | C06414 | Up | 0.02 | 0.43 |
| Feces | O-Methylthreonine | NA | 2724875 | NA | Down | 0.03 | 0.17 |
| Feces | Caffeic Acid | HMDB0001964 | 1549111 | C01481 | Up | 0.03 | 0.76 |
| Feces | 2-Deoxyuridine | HMDB0000012 | 13712 | C00526 | Up | 0.04 | 0.64 |
| Feces | Methyl Icosanoate | NA | 14259 | NA | Down | 0.03 | 0.18 |
| Feces | Epsilon-Caprolactam | METPA0843 | 7768 | C06593 | Up | 0.01 | 0.48 |
| Feces | 5-Hydroxytryptophan | HMDB0000472 | 144 | C00643 | Up | 0.03 | 0.35 |
| Feces | Trans-4-Hydroxy-L-Proline | HMDB0000725 | 5810 | C01157 | Up | 0.03 | 0.91 |
| Serum | Palmitic Acid | HMDB0000220 | 985 | C00249 | Down | 0.04 | 0.99 |
| Serum | 2-Ketobutyric Acid | HMDB0000005 | 58 | C00109 | Up | 0.00 | 0.53 |
| Serum | 2-Hydroxybutanoic Acid | HMDB0000008 | 11266 | C05984 | Down | 0.00 | 0.22 |
| Serum | Proline | HMDB0000162 | 145742 | C00148 | Up | 0.00 | 0.97 |
| Serum | Alanine | HMDB0000161 | 5950 | C00041 | Up | 0.02 | 0.93 |
| Serum | Inosine | HMDB0000195 | 6021 | C00294 | Down | 0.00 | 0.55 |
| Serum | Myo-Inositol | HMDB0000211 | 892 | C00137 | Up | 0.00 | 0.17 |
| Serum | 3-Hydroxybutyric Acid | HMDB0000357 | 441 | C01089 | Down | 0.00 | 0.53 |
| Serum | Valine | HMDB0000883 | 6287 | C00183 | Up | 0.00 | 0.97 |
| Serum | Glucose | HMDB0000122 | 5793 | C00221 | Down | 0.02 | 0.53 |
| Serum | Beta-Glycerophosphoric Acid | HMDB0002520 | 2526 | C02979 | Down | 0.00 | 0.97 |
| Serum | D-Talose | NA | 441035 | C06467 | Up | 0.00 | 1.00 |
| Hippocampus | Fumaric Acid | HMDB0000134 | 444972 | C00122 | Down | 0.04 | 0.06 |
| Hippocampus | 3-Hydroxybutyric Acid | HMDB0000357 | 441 | C01089 | Down | 0.00 | 0.05 |
| Hippocampus | Palmitic Acid | HMDB0000220 | 985 | C00249 | Up | 0.01 | 0.26 |
| Hippocampus | Dihydroxyacetone | HMDB0001882 | 670 | C00184 | Down | 0.03 | 0.91 |
| Hippocampus | 4-Aminobutyric Acid | HMDB0000112 | 223130 | C00334 | Down | 0.03 | 0.13 |
| Hippocampus | Glycerol | HMDB0000131 | 753 | C00116 | Down | 0.00 | 0.42 |
| Hippocampus | Ascorbate | NA | 3372 | C00072 | Down | 0.05 | 0.76 |
| Prefrontal Cortex | 1-Hexadecanol | HMDB0003424 | 2682 | C00823 | Up | 0.00 | 0.93 |
| Prefrontal Cortex | Lactic Acid | HMDB0000190 | 61503 | C00186 | Up | 0.00 | 0.95 |
| Striatum | Dithioerythritol | HMDB0251493 | 439352 | C00950 | Down | 0.02 | 0.30 |
| Striatum | Mannitol | HMDB0000765 | 6251 | C00392 | Up | 0.01 | 0.77 |
| Striatum | Inosine | HMDB0000195 | 6021 | C00294 | Down | 0.03 | 0.79 |
| Striatum | 3-Hydroxybutyric Acid | HMDB0000357 | 441 | C01089 | Down | 0.00 | 0.94 |
| Striatum | Hydroxylamine | HMDB0003338 | 787 | C00192 | Down | 0.03 | 0.14 |
| Striatum | Creatine Degr | NA | NA | NA | Down | 0.01 | 0.79 |

**Supplementary table 2. The VIP values of all reversed metabolites found in HMb and MMb groups.**

| **Sample** | **Reversed Metabolites** | **VIP** | **Sample** | **Reversed Metabolites** | **VIP** |
| --- | --- | --- | --- | --- | --- |
| Feces | Sinapyl alcohol | 1.95 | Feces | Caffeic acid | 1.20 |
| Feces | Alpha-Santonin | 1.85 | Feces | 2-Deoxyuridine | 1.17 |
| Feces | Fumaric acid | 1.73 | Feces | L-4-Hydroxyphenylglycine | 1.16 |
| Feces | 2,3-Dimethylsuccinic acid | 1.72 | Feces | Phenylacetaldehyde | 1.16 |
| Feces | 4-Hydroxy-3-methoxybenzoic acid | 1.70 | Feces | Luteolin | 1.11 |
| Feces | D-(glycerol-phosphate) | 1.68 | Serum | 3-Hydroxybutyric acid | 1.68 |
| Feces | Maltitol | 1.68 | Serum | D-Talose | 1.64 |
| Feces | Formononetin | 1.65 | Serum | Inosine | 1.64 |
| Feces | Tartronic acid | 1.65 | Serum | Myo-inositol | 1.57 |
| Feces | N-Acetyl-L-glutamic acid | 1.64 | Serum | Valine | 1.55 |
| Feces | 4-oxo-1H-quinoline-2-carboxylic acid | 1.63 | Serum | 2-Hydroxybutanoic acid | 1.51 |
| Feces | 3,4-Dihydroxyphenylglycol | 1.61 | Serum | Beta-glycerophosphoric acid | 1.41 |
| Feces | 3-Hydroxybutyric acid | 1.60 | Serum | 2-Ketobutyric acid | 1.41 |
| Feces | 1-Hydroxyanthraquinone | 1.59 | Serum | Proline | 1.35 |
| Feces | Phosphate | 1.57 | Serum | Palmitic acid | 1.27 |
| Feces | L-dopa | 1.53 | Serum | Glucose | 1.21 |
| Feces | Putrescine | 1.50 | Serum | Alanine | 1.21 |
| Feces | Threonic acid | 1.48 | Hippocampus | 3-Hydroxybutyric acid | 2.23 |
| Feces | Beta-glycerophosphoric acid | 1.47 | Hippocampus | Glycerol | 1.98 |
| Feces | Daidzein | 1.47 | Hippocampus | Ascorbate | 1.94 |
| Feces | Cytidine | 1.45 | Hippocampus | Palmitic acid | 1.76 |
| Feces | Methyl icosanoate | 1.44 | Hippocampus | Fumaric acid | 1.59 |
| Feces | Succinic acid | 1.41 | Hippocampus | Dihydroxyacetone | 1.55 |
| Feces | Quinoline-4-carboxylic acid | 1.40 | Hippocampus | 4-Aminobutyric acid | 1.50 |
| Feces | Erythrose | 1.39 | Prefrontal cortex | 1-Hexadecanol | 1.94 |
| Feces | Beta-alanine | 1.39 | Prefrontal cortex | Lactic acid | 1.65 |
| Feces | N-Acetyl-beta-alanine | 1.34 | Striatum | 3-Hydroxybutyric acid | 1.93 |
| Feces | O-methylthreonine | 1.33 | Striatum | Creatine degr | 1.53 |
| Feces | N-ethylmaleamic acid | 1.32 | Striatum | Dithioerythritol | 1.45 |
| Feces | Uridine | 1.31 | Striatum | Hydroxylamine | 1.43 |
| Feces | Lactic acid | 1.28 | Striatum | Mannitol | 1.39 |
| Feces | 5-Hydroxytryptophan | 1.28 | Striatum | Inosine | 1.36 |
| Feces | Trehalose-6-phosphate | 1.27 |  |  |  |
| Feces | Homovanillic acid | 1.27 |  |  |  |
| Feces | Trans-4-hydroxy-l-proline | 1.26 |  |  |  |
| Feces | Levoglucosan | 1.25 |  |  |  |
| Feces | 2-Ketocaproic acid | 1.23 |  |  |  |
| Feces | Epsilon-caprolactam | 1.22 |  |  |  |
| Feces | Gallic acid | 1.22 |  |  |  |

**Supplementary table 3. Correlation between** **the abundance of reversed gut microbes and anxiety like behavior.**

| **Phyla** | **Family** | **OTU** | **Central_distance** | **P** | **Cenral_time** | **P** | **Expression in MMb** |
| --- | --- | --- | --- | --- | --- | --- | --- |
| Firmicutes | Peptostreptococcaceae | OTU270 | -0.433 | 0.039 | -0.336 | 0.117 | Up |
| Firmicutes | Ruminococcaceae | OTU3210 | 0.427 | 0.042 | 0.274 | 0.206 | Down |
| Firmicutes | Christensenellaceae | OTU3256 | 0.387 | 0.068 | 0.631 | **0.001** | Down |
| Firmicutes | Lactobacillaceae | OTU3266 | -0.495 | 0.016 | -0.376 | 0.077 | Up |
| Firmicutes | Lachnospiraceae | OTU3615 | -0.430 | 0.041 | -0.285 | 0.188 | Up |
| Firmicutes | Erysipelotrichaceae | OTU3272 | -0.450 | 0.031 | -0.355 | 0.096 | Up |
| Bacteroidetes | Bacteroidaceae | OTU3268 | -0.546 | **0.007** | -0.39 | 0.065 | Up |
| Bacteroidetes | Bacteroidaceae | OTU3288 | -0.636 | **0.001** | -0.463 | 0.026 | Up |
| Bacteroidetes | Bacteroidaceae | OTU3436 | -0.554 | **0.006** | -0.443 | 0.034 | Up |
| Bacteroidetes | Porphyromonadaceae | OTU3278 | -0.453 | 0.03 | -0.347 | 0.105 | Up |
| Bacteroidetes | Porphyromonadaceae | OTU3304 | -0.460 | 0.027 | -0.335 | 0.118 | Up |
| Bacteroidetes | Porphyromonadaceae | OTU3629 | -0.462 | 0.026 | -0.352 | 0.099 | Up |
| Bacteroidetes | Rikenellaceae | OTU3310 | 0.594 | **0.003** | 0.585 | **0.003** | Down |
| Verrucomicrobia | Verrucomicrobiaceae | OTU3262 | 0.429 | 0.041 | 0.252 | 0.247 | Down |
| Unclassified_k__norank | Unclassified_k__norank | OTU3169 | 0.555 | **0.006** | 0.364 | 0.088 | Down |

P values of < 0.05 was considered statistically significant.
